# A highly thermostable crude endoglucanase produced by a newly isolated *Thermobifida fusca* strain UPMC 901

**DOI:** 10.1038/s41598-019-50126-y

**Published:** 2019-09-19

**Authors:** Mohd Huzairi Mohd Zainudin, Nurul Asyifah Mustapha, Mohd Ali Hassan, Ezyana Kamal Bahrin, Mitsunori Tokura, Hisashi Yasueda, Yoshihito Shirai

**Affiliations:** 1 Laboratory of Sustainable Animal Production and Biodiversity, Institute of Tropical Agriculture and Biodiversity, Universiti Putra Malaysia, 43400 Serdang, Selangor Malaysia; 2 Department of Biological Function and Engineering, Graduate School of Life Science and System Engineering, Kyushu Institute of Technology, 2-4 Hibikino-cho, Wakamatsu-ku, Fukuoka, 808-0196 Japan; 3 Department of Bioprocess Technology, Faculty of Biotechnology and Biomolecular Sciences, Universiti Putra Malaysia, 43400 UPM Serdang, Selangor Malaysia; 4 Advanced Microbiological Functions Research Group, Frontier Research Labs., Institute for Innovation, Ajinomoto, 1-1 Suzuki-cho, Kawasaki-ku, Kawasaki, Japan

**Keywords:** Applied microbiology, Biocatalysis

## Abstract

A thermophilic *Thermobifida fusca* strain UPMC 901, harboring highly thermostable cellulolytic activity, was successfully isolated from oil palm empty fruit bunch compost. Its endoglucanase had the highest activity at 24 hours of incubation in carboxymethyl-cellulose (CMC) and filter paper. A maximum endoglucanase activity of 0.9 U/mL was achieved at pH 5 and 60 °C using CMC as a carbon source. The endoglucanase properties were further characterized using crude enzyme preparations from the culture supernatant. Thermal stability indicated that the endoglucanase activity was highly stable at 70 °C for 24 hours. Furthermore, the activity was found to be completely maintained without any loss at 50 °C and 60 °C for 144 hours, making it the most stable than other endoglucanases reported in the literature. The high stability of the endoglucanase at an elevated temperature for a prolonged period of time makes it a suitable candidate for the biorefinery application.

## Introduction

Lignocellulosic biomass, earth’s most abundant renewable material, consisting of cellulose, hemicellulose and lignin, are naturally degraded by many lignocellulolytic microorganisms including bacteria and fungi. The degradation of this biomass, particularly cellulose, is accomplished by the synergistic action of three types of enzymes, namely endoglucanase, exoglucanase and β-glucosidase^1^. These enzymes are important in order to completely depolymerize cellulose into glucose. Endoglucanases and exoglucanases act synergistically on cellulose to form short-chain oligosaccharides and cellobiose which are then broken down into glucose by β-glucosidase.

Cellulases with high thermal stability and optimum activity at elevated temperature have received great interest in various industrial applications such as food, textile, pulp, paper industry and agro-industrial biomass conversion. This is due to its potential advantages such as shorter hydrolysis period by the high specific activity of the enzyme, better solubility of the substrate/products, lower risk of contamination in the degradation process and lower cost of energy for the cooling process after the pre-treatment^2,3^. One of the most essential criteria for the enzymes to be employed in the industry is that the protein should be strong enough and highly stable to withstand the severe condition of biorefinery processes like extreme temperature. Maintaining most of the activity of the enzymes for at least the duration of the biorefining process is also important especially when the process needs to be completed in a few days. In addition to the thermal stability, the use of crude cellulases is also considered advantageous as compared to the purified form. Besides, the crude cellulase consists of additional enzyme activities which may improve the enzymatic hydrolysis of cellulosic material^4^. It has been reported that the yield of sugars obtained from the hydrolysis of sugarcane bagasse is comparable to that of the commercial cellulase, indicating that impurities do not influence the activity of the crude cellulase^5^.

Not only that thermophilic bacterium can survive under extreme condition, they also produce stable cellulases which may improve the bioconversion process^2,6^. Recently, several thermophilic strains producing thermostable endoglucanases have been isolated and identified from various environments such as compost^7,8^, sugar refinery waste^9^, deep subsurface of the former gold mine^10^, decaying straw, leaves and switchgrass^11^. These isolated thermophilic bacteria include some species from *Firmicutes* phylum which was mainly *Bacillus* sp.^2^ and also from *Actinobacteria* such as *Thermobifida* sp. or previously known as *Thermomonospora* sp. and *Streptomyces* sp.^12–16^. During the composting process, the degradation of lignocellulosic materials mainly occurs during the sustained thermophilic phase^17,18^. In our previous study, several cellulolytic bacteria had been successfully isolated from different stages of the oil palm empty fruit bunch composting process, of which the thermophilic stage had the highest cellulolytic bacterial population as compared to other stages^19^. These findings indicated that the presence of various cellulolytic bacteria, especially during the thermophilic stage, serves as one of the best sources for discovering the thermostable cellulose degrading enzymes. Therefore, this study aimed to evaluate the production as well as the thermostable properties of the crude endoglucanase produced by the isolated *Thermobifida fusca* UPMC 901.

## Results and Discussion

### The cellulolytic strain

**Figure 1:**
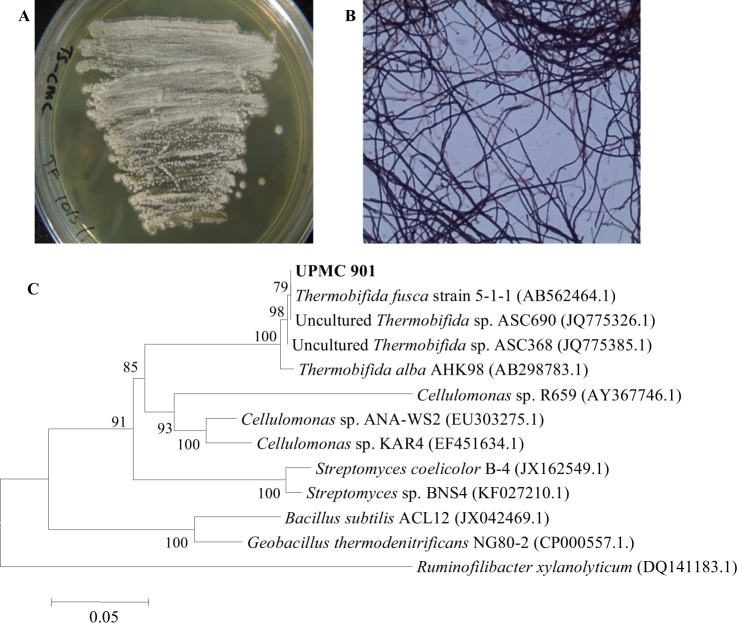
Identification of isolated *T. fusca* UPMC 901 strain. **(A)** Strain cultivated on TS medium agar plate containing 0.2% (w/v) CMC after incubating at 50 °C for 48 hours. **(B)** Morphological view of a gram-stained *T. fusca* UPMC 901 strain under a light microscope at 400X magnification. **(C)** Phylogenetic analysis of 16S rRNA sequence of the *T. fusca* UPMC 901 strain with the sequences of *Thermobifida* type strains from the Genebank using neighbor-joining method. The bar represents 0.05 substitutions per nucleotide position and numbers at the node are the bootstrap values (%). *Ruminofilibacter xylanolyticum* (DQ141183.1) was used as an outgroup.

During the composting process, the degradation of lignocellulosic material started during the thermophilic phase after which the readily decomposable organic materials have been consumed by the microbes. Some of the bacteria will produce and release cellulases in order to convert the complex lignocellulosic structure into simple sugars. In this study, a gram-positive, spore-forming, filamentous, thermophilic cellulolytic bacterium UPMC 901 was successfully isolated during the thermophilic stage of the OPEFB composting process (Fig. 1A,B). The UPMC 901 strain has the ability to express higher endoglucanase activity within 24 hours of incubation as compared to the other strains which took 48 hours to produce higher endoglucanase activity (Table 1). In addition, their activities were lower than the UPMC 901. The 16S rRNA analysis of UPMC 901 strain showed that its sequence is related to both cultured and uncultured *T. fusca* (Fig. 1C). The Blast-N algorithm results of 16S rDNA sequence similarity of isolated UPMC 901 also showed that this bacterium was closely related to cultured and uncultured *Thermobifida* species with 99% similarity (Table 2). As reported earlier, *T. fusca* is a filamentous soil thermophilic bacterium that grows at 50–55 °C in tryptic soy medium on cellulose as a carbon source. This bacterium has an endospore, which is necessary to survive in the extreme environment for prolonged periods of time and usually grows as aerial hyphae which help the bacteria to penetrate and decompose cellulose^15^. *T. fusca* produces several cellulolytic enzymes including exoglucanases, endoglucanases, β-glucosidase, β-cellobiosidase and appears to be a major degrader of a plant cell walls in heated organic materials such as compost pile and rotting hay^16^. Moreover, *T. fusca* is a well-known cellulolytic bacterium having high cellulase activity such as endoglucanases with tolerance to a broad range of pH and thermal stability^15^. Nevertheless, the thermal stability properties of these cellulases may vary between each *Thermobifida* species. Based on the rapid production, higher endoglucanase activity and phylogenetically affiliated with uncultured *T. fusca* species, it is suggested that the UPMC 901 could be an effective strain of the genus *Thermobifida* which might exhibit unique endoglucanase properties. Hence, in this study, the production as well as the thermal stability of endoglucanase from isolated *T. fusca* strain UPMC 901, was further evaluated.

**Table 1:** Endoglucanase activity of thermophilic bacterial strains isolated during the composting process.

Strain Accession ID	Incubation temperature	Incubation time	Maximum Endoglucanase activity (U/mL)	
			Filter paper	CMC
UPMC 901	50 °C	24 hours	0.18	0.50
UPMC 902		48 hours	0.05	0.10
UPMC 903		48 hours	0.08	0.10
UPMC 904		48 hours	0.18	0.24

**Table 2:** The Blast-N results of the UPMC 901 strain 16S rRNA gene.

Species	Maximum score	Query cover (%)	Maximum Identity (%)	Accession Number
Uncultured *Thermobifida* sp. clone ASC690	1328	100	99	JQ775326.1
*Thermobifida fusca* strain: 5-1-1	1328	100	99	AB562464.1
Uncultured *Thermobifida* sp. clone ASC368	1323	100	99	JQ775385.1
*Thermobifida alba* strain, AHK98	1206	100	97	AB298783.1

### Endoglucanase activity on different carbon sources

**Figure 2:**
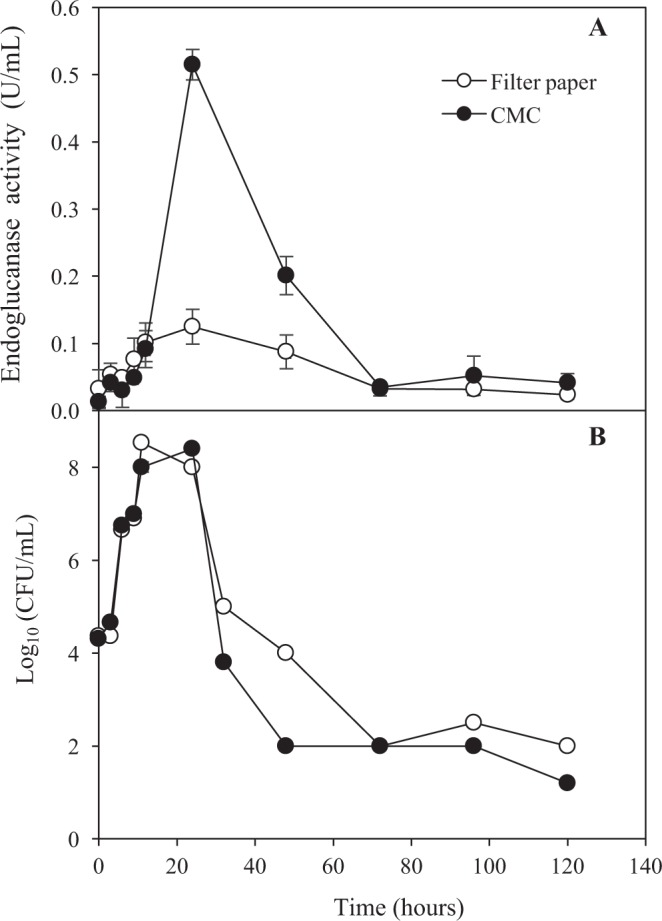
(**A**) Endoglucanase activity of *T. fusca* UPMC 901 on different carbon sources (FP: Filter paper and CMC: Carboxymethyl-cellulose) incubated for 120 hours at 50 °C. (**B**) The growth of *T. fusca* UPMC 901 measured by the number of colony form unit (CFU) per milliliter of culture supernatant grown with filter paper and CMC. The standard deviations for the mean of three replicates are shown as error bars.

Carbon source plays an important role in inducing endoglucanase activity during the growth phase of the cellulolytic bacterium^6^. As shown in Fig. 2A, the endoglucanase activity of the crude enzyme supernatant from liquid culture was evaluated in which CMC or FP was used as carbon sources, respectively. The results showed that the crude enzyme of CMC medium had higher endoglucanase activity as compared to FP. Higher endoglucanase activity achieved was attributed to the easily hydrolyzable amorphous structure of CMC. Meanwhile, FP which mainly consists of a crystalline structure of α-cellulose makes this material less susceptible to the enzymatic hydrolysis. A similar result was obtained through the cultivation of *Bacillus* sp VG1 in CMC, whereby higher production of endoglucanase was achieved compared to cellulose and newspaper^20^. Previous studies reported that thermophilic *Streptomyces* transformant T3-1 strains, which was isolated from the compost, produced endoglucanase with the highest enzyme activity after 48 hours of incubation at 50 °C in 1% (w/v) of CMC medium^7^. The maximum endoglucanase activity of *T. fusca* UPMC 901 was achieved after 24 hours of fermentation in both media as compared to the previous studies including the other 3 isolates (UPMC 902, UPMC 903 and UPMC 904) of this study. Thus, it could be demonstrated that *T. fusca* UPMC 901 appeared to be a potential strain for rapid production of the endoglucanase, which may be advantageous to the biorefinery processes. Figure 2B shows the growth profile of the bacterium throughout the fermentation. It can be seen from the figure that the endoglucanase was being produced during the growth phase of the *T. fusca* UPMC 901. This result is in agreement with the previous study which reported that the endoglucanase production was associated with the increase in total cellular protein during the active growth of the bacterium^8^.

### Characteristics of the crude endoglucanase

**Figure 3:**
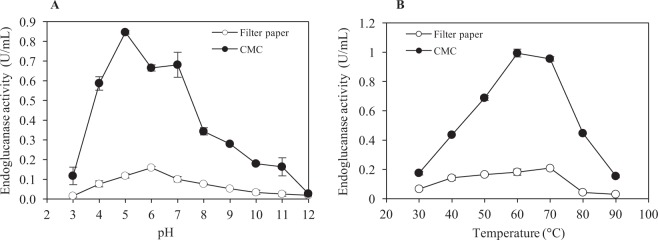
The effect of temperature **(A)** and pH **(B)** on endoglucanase activity of the crude enzyme produced by *T. fusca* UPMC 901 strain using filter paper (FP) and carboxymethyl-cellulose (CMC) as substrates. Determination of optimum temperature on endoglucanase activity was done in 0.05 M sodium citrate buffer at pH 6 and incubated for 30 min. The determination of optimum pH was done by incubating the enzyme at different pH buffer (3–12) at 50 °C for 30 min. The standard deviations for the mean of three replicates are shown as error bars.

**Figure 4:**
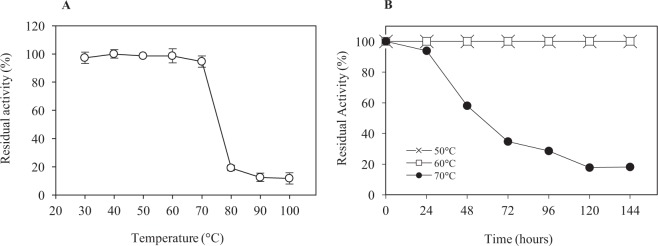
The effect of temperature **(A)** and time **(B)** on endoglucanase stability of crude enzyme preparation obtained from CMC cell-free culture supernatant of *T. fusca* UPMC 901. The optimum temperature was determined by incubating the reaction mixture at a different temperature at optimum pH 5 for 1 hour, whereas to determine the thermal stability of endoglucanase, the crude enzyme solution was stored at different temperature (50 °C, 60 °C and 70 °C) for 144 hours. The standard deviations for the mean of three replicates are shown as error bars.

**Figure 5:**
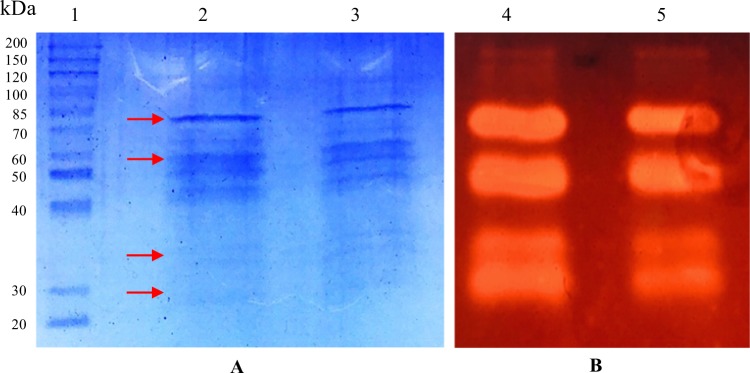
Protein profile of the *T. fusca* UPMC 901 crude enzyme preparation (**A**) and activity of the crude enzyme visualized by using SDS gel containing 0.5% (w/v) of CMC (**B**). Lane 1: 150 kDa Marker; Lane 2 and 3- Duplicates of extracellular proteins of CMC cell-free culture supernatant: Lane 4 and 5 – endoglucanase activity (CMCase). The arrows indicate candidate protein bands for the *T. fusca* UPMC 901 endoglucanase. The original image of SDS-PAGE and Zymogram gel were displayed in Supplementary Fig. S1 and S2.

The effect of pH and temperature on endoglucanase activity of crude enzyme supernatant obtained from CMC and FP medium is shown in Fig. 3A,B. The maximum endoglucanase activity was achieved at pH 5 for CMC (0.85 U/mL) and pH 6 for FP (0.16 U/mL), respectively. The difference in the pH tested suggests the sorption of filter paper surface unfolded the structure of the enzymes on the solid surface which increased its optimum pH by one pH unit of the crude enzyme extract of FP medium^21^. The highest endoglucanase activity was recorded at 60 °C and 70 °C in a liquid culture containing CMC (0.99 U/mL) and FP (0.21 U/mL), respectively. It has been reported in an earlier study that *T. fusca* exhibited the maximum endoglucanase activity at the temperature between 40–70 °C in the broad pH range (5–10)^15^. The effect of temperature and time on endoglucanase activity of the crude enzyme obtained from the CMC medium was further evaluated. The results of endoglucanase activity assays showed that the enzyme retained 94% of the original activity up to 70 °C (Fig. 4A). The endoglucanase activity decreased abruptly with only 11–19% of the original activity retained when the enzyme was incubated at 80 °C, 90 °C and 100 °C for 1 hour. Thermal stability profile revealed that the endoglucanases of *T. fusca* UPMC 901 completely retained 100% of initial activity during the incubation at 50 °C and 60 °C for 144 hours (Fig. 4B). SDS-PAGE and zymogram analysis were performed to observe extracellular endoglucanase protein of the crude extract obtained from the growth medium. The SDS-PAGE gel showed the bands with a molecular weight ranging from 85 kDa to 30 kDa (Fig. 5A). The zymogram analysis shows 4 major candidate proteins bands of endoglucanase (CMCase) with the size of 80 kDa, 60 kDa, 35 kDa and 30 kDa bands actively hydrolyzed the CMC which was supplemented into the gel (Fig. 5B). A previous study described that other *T. fusca* strains have endoglucanases, which have a similar molecular weight distribution (90.4 kDa to 30.4 kDa)^22^. Overall, SDS-PAGE and zymograms provided a detailed insight on cell content of *T. fusca* UPMC 901 showing it produces a series of endoglucanases enzymes which were important for hydrolyzing the cellulosic materials.

In comparison with the other thermostable endoglucanases of which the activity reduced within 24 hours or less in the temperature range of 50 °C-60 °C, the endoglucanase activity of *T. fusca* UPMC 901 strain was found to be completely stable for a longer period of time (Table 3). Most of the reported endoglucanases were also found to be less stable at 70 °C. However, our present study showed that the *T. fusca* UPMC 901 endoglucanase was able to maintain 94% of its initial activity at 70 °C for 24 hours. An earlier study by Posta *et al*.^16^ reported that the endoglucanase Cel5B of *T. fusca* exhibited a residual activity of less than 20% when the enzyme was incubated at 70 °C for 24 hours, which is lower than this study. The results of this study indicated that the endoglucanase is highly stable at elevated temperature with a prolonged period of time, thus making it the most thermostable endoglucanase among the reports to date. Highly thermostable endoglucanases obtained could be due to the fact that the enzyme is able to sustain its activity at the thermophilic stage, during which the temperature of the compost pile was maintained at 50–60 °C for 20 days. In addition, it has been reported that the external environmental factors such as polyols, sugars, and protein increased the thermal stability of the crude enzyme^23,24^.

**Table 3:** Comparison of thermal stability of crude endoglucanase produced by thermophilic cellulolytic bacteria between this study and the literature.

Strain	Source of isolation	Enzyme production condition (Medium Substrate, Temperature, rpm)	Stability of crude endoglucanase						References
			50 °C	Time (hour)	60 °C	Time (hour)	70 °C	Time (hour)	
*Geobacillus thermoloevorans* T4	Sugar refinery wastewater	Mendels medium, CMC, 60 °C, 120 rpm	98%	1	98%	1	97%	1	^9^
*Bacillus* sp. SM1A-2	Soil from Campos dos Goytacazes city,	Minimal medium, baggase, 50 °C, 150 rpm	100%	1	100%	1	50%	1	^29^
*Streptomyces transformant* T3-1	Agricultural waste compost	Mendels medium, CMC, 50 °C, 150 rpm	100%	5	100%	5	77%	5	^7^
*Bacillus sp*. VGI	Hot spring	Minimal medium, CMC, 45 °C150 rpm	100%	12	70%	12	40%	12	^20^
*Bacillus* sp. WSCUF1	Organic waste compost	Minimal medium, cellulose, 60 °C, 120 rpm	100%	24	100%	24	89%	24	^8^
*Geobacillus* sp. DUSELR7	Deep surface of the homestake gold mine	Minimal medium, cellulose, 60 °C, 120 rpm	ND	—	97%	24	70%	24	^10^
*Thermomonospora curvata*	ND	Minimal medium, cotton fiber, 53 °C	ND	—	100%	1	100%	1	^30^
*Thermomonospora* sp.	ND	Minimal medium, cellulose, 55 °C, 170 rpm	100%	24	100%	24	50%	24	^31^
*Thermobifida fusca* UPMC 901	Oil palm empty fruit bunch compost	Tryptic soy medium, CMC, 50 °C, 150 rpm	100%	144	100%	144	94%	24	**This study**

ND: Not determined.

## Materials and Method

### Strains, medium and culture conditions

A total of 4 thermophilic cellulolytic strains (UPMC 901, UPMC 902, UPMC 903, UPMC 904) which were isolated from oil palm empty fruit bunch compost heap and deposited at Culture Collection Unit (UNiCC), Institute of Bioscience, Universiti Putra Malaysia were grown on the Tryptic Soy (TS) or Luria Bertani (LB) agar plate containing 0.2% (w/v) carboxymethylcellulose (CMC) at 50 °C. The strains were previously screened for the cellulolytic activity by growing the bacterium on the agar plate containing 0.2% (w/v) CMC. The strain harboring endoglucanase activity showed a clear zone around the colony on the plate after staining with 1% (w/v) Congo red^19^. The endoglucanase activity of the strains was further evaluated in liquid culture condition by growing the bacterium, in a TS medium containing yeast extract (0.5 g/L) and each 1% (w/v) of filter paper (FP) and CMC as a carbon source^25,26^. The media (100 mL) were then inoculated with 10% (v/v) of bacterial suspension (approximately with 1.0 optical density of cell at 660 nm). The cultures were incubated at 150 rpm for 24 hours at 50 °C. The cell-free culture supernatant was prepared by centrifugation at 5000 × *g* for 15 minutes at 4 °C. The culture supernatant was then used for analysing the endoglucanase activity.

### Identification of strain

The identification of bacteria was done through PCR colony method using a set of reverse and forward primers; 27F (5′-AGA GTT TGA TCC TGG CTC AG-3′) and 1492R (5′-GGT TAC CTT GTT ACG ACT T-3′). The amplification conditions of 16S rRNA gene were carried out according to the method described in our previous study^19^. The PCR product was then purified using a PCR purification kit (Yeastern Biotech, Taiwan). The amplified products were sequenced and analysed in the National Center for Biotechnology Information (NCBI) using the nucleotide-nucleotide Basic Local Alignment Search online tool (BLASTn; http://blast.ncbi.nlm.gov/Blast.cgi) program. The phylogenetic tree was constructed using MEGA 4.0 software through the neighbor-joining method. The nucleotide sequence was deposited under accession number KF305097 in the Genebank database. The morphological characteristic of the bacterium was determined by light microscope (Olympus CX21, Tokyo, Japan). In addition, the gram stain of this strain was identified by treating the cell with the gram staining solutions according to the method described by Benson^27^.

### Evaluation of endoglucanase activity on the different carbon sources

The bacteria strain with the highest endoglucanase activity (UPMC 901) was grown in the 150 mL of TS medium containing each 1% (w/v) CMC and FP as the carbon sources. Control without substrate was also included in the experiment. The culture (5 mL) was collected at 0, 3, 6, 12, 24, 48, 72, 96, and 120 hours. The culture was then centrifuged at 5000 × *g* for 15 minutes at 4 °C and the supernatant was analysed for the endoglucanase activity.

### Endoglucanase activity assay and characterization of crude endoglucanase

Endoglucanase activity of the crude endoglucanase obtained from the cell-free culture supernatant of both (CMC and FP) medium which was used as a carbon source, was assayed using the method described by Ariffin *et al*.^28^. The reaction mixture consisted of 1.8 mL of 0.05 M sodium citrate buffer (pH 4.8) containing 1% (w/v) CMC and 0.2 mL crude enzyme solution. The reaction mixture was incubated at 50 °C for 30 minutes. The reaction was then terminated by adding 3 mL of DNS reagent and incubated at 100 °C for 15 minutes. After cooling, 1 mL of Rochelle’s salt was added for color stabilization. Substrate and enzyme blank were included in all assays. The absorbance was recorded at 575 nm and the amount of reducing sugar was estimated against a glucose standard curve. Endoglucanase activity was measured by determining the amount of reducing sugar released from CMC. One unit of endoglucanase activity was defined as the amount of enzyme that released 1 µmol of glucose equivalent per minute. The optimum pH for the activity of the crude endoglucanase was investigated by carrying out the reaction in 0.05 M sodium citrate buffer at the range of pH 3–7 and 0.05 M glycine-NaOH buffer at the range of pH 8–12, which was done at 50 °C. The optimum pH of 5 was used to determine the optimum temperature for the crude endoglucanase activity. The optimal temperature for crude endoglucanase was determined by performing the endoglucanase assays at different temperatures (30–90 °C). The effect of temperature on endoglucanase stability was determined by incubating the crude enzyme at the temperature ranging from 30–100 °C for 1 hour. The effect of time on the thermal stability of the enzyme was then evaluated by determining the residual activity after incubation of the crude enzyme solution at 50 °C, 60 °C and 70 °C for a period of 6 days. Initial residual activity was assumed to be 100% and was used to compare with the enzyme activities obtained during the incubation period.

### Sodium dodecyl sulfate polyacrylamide gel electrophoresis (SDS-PAGE) and zymogram

The SDS-PAGE analysis was conducted using 12% (w/v) polyacrylamide gel. The crude enzyme (22.5 μL) was prepared by mixing with 7.5 μL of 4 × SDS-based sample loading buffer and heated at 100 °C for 5 min. The electrophoresis was conducted at 90 V for 90 min. The gel was immersed in 0.1% (w/v) Coomassie Brilliant Blue solution for 60 min followed by destaining with a solution containing 25% (v/v) methanol and 7% (v/v) acetic acid. Zymogram analysis for endoglucanase was performed by incorporating 0.5% (w/v) of CMC into polyacrylamide gel. After separation, the gel was stained using Coomassie Brilliant Blue dye. Another set of gels with enzymes was washed for 2 times, each for 30 min with 2.5% (v/v) Triton-X in 50 mM sodium citrate buffer at room temperature to remove SDS and re-nature the enzyme components. The enzymes were allowed to react with substrate incorporated into the gel by incubating in 50 mM sodium acetate buffer at 50 °C for 1 hour. The gel was stained using 1% (w/v) Congo red solution for 30 min and destained using 1 M NaCl for 3 times each for 30 min. The active bands for endoglucanases were shown as clear hydrolysis bands against the dark red background.

## Conclusion

Due to the increasing demand for more stable cellulases such as endoglucanases in the biorefinery processes, an enzyme with highly thermostable properties is necessarily required. Our current study showed that a cellulolytic *T. fusca* UPMC 901 strain isolated during the thermophilic stage of composting process expressed higher endoglucanase activity at 24 hours of incubation when CMC was used as the carbon source compared to the filter paper. Interestingly, the endoglucanases were found to be stable during the prolonged period of time (144 hours) at 50 °C and 60 °C. The endoglucanases of *T. fusca* UPMC 901 are also able to maintain 94% of its activity at 70 °C for 24 hours in comparison to other thermostable endoglucanases previously reported. Thus, the thermostable crude endoglucanase of *T. fusca* UPMC 901 has a great potential to be employed in the biorefinery applications.

## Supplementary information

Supplementary dataset
