# Author Correction: A highly thermostable crude endoglucanase produced by a newly isolated *Thermobifida fusca* strain UPMC 901

**DOI:** 10.1038/s41598-020-58488-4

**Published:** 2020-01-27

**Authors:** Mohd Huzairi Mohd Zainudin, Nurul Asyifah Mustapha, Mohd Ali Hassan, Ezyana Kamal Bahrin, Mitsunori Tokura, Hisashi Yasueda, Yoshihito Shirai

**Affiliations:** 1 Laboratory of Sustainable Animal Production and Biodiversity, Institute of Tropical Agriculture and Food Security, Universiti Putra Malaysia, 43400 Serdang, Selangor Malaysia; 2 Department of Biological Function and Engineering, Graduate School of Life Science and System Engineering, Kyushu Institute of Technology, 2-4 Hibikino-cho, Wakamatsu-ku, Fukuoka, 808-0196 Japan; 3 Department of Bioprocess Technology, Faculty of Biotechnology and Biomolecular Sciences, Universiti Putra Malaysia, 43400 UPM Serdang, Selangor Malaysia; 4 Advanced Microbiological Functions Research Group, Frontier Research Labs., Institute for Innovation, Ajinomoto, 1-1 Suzuki-cho, Kawasaki-ku, Kawasaki, Japan

Correction to: *Scientific Reports* 10.1038/s41598-019-50126-y, published online 19 September 2019

The original version of this Article contained an error in Affiliation 1, which was incorrectly given as ‘Laboratory of Sustainable Animal Production and Biodiversity, Institute of Tropical Agriculture and Biodiversity, Universiti Putra Malaysia, 43400, Serdang, Selangor, Malaysia’. The correct affiliation is listed below:

Laboratory of Sustainable Animal Production and Biodiversity, Institute of Tropical Agriculture and Food Security, Universiti Putra Malaysia, 43400, Serdang, Selangor, Malaysia.

Additionally, there was a typographical error in Table 3, where ‘Geobacillus thermoloevorans T4’ now reads ‘Geobacillus thermoleovorans T4’.

These errors have now been corrected in the HTML and PDF versions of the Article, and the accompanying Supplementary information.

