# Supplementary material for: A highly thermostable crude endoglucanase produced by a newly isolated Thermobifida fusca strain UPMC 901
Source: Sci Rep. 2019 Sep 19;9:13526. doi: 10.1038/s41598-019-50126-y (PMC6753106; doi:10.1038/s41598-019-50126-y)
Supplement: Supplementary file 1 — Supplementary dataset [file 41598_2019_50126_MOESM1_ESM.docx]

**SUPPLEMENTARY MATERIALS**

**Journal:** Scientific Reports

**A highly thermostable crude endoglucanase produced by a newly isolated *Thermobifida fusca* strain UPMC 901**

^1^Mohd Huzairi Mohd Zainudin*, ^2^Nurul Asyifah Mustapha, ^3^Mohd Ali Hassan, ^3^Ezyana Kamal Bahrin, ^4^Mitsunori Tokura, ^4^Hisashi Yasueda, ^2^Yoshihito Shirai

^1^Laboratory of Sustainable Animal Production and Biodiversity, Institute of Tropical Agriculture and Biodiversity, Universiti Putra Malaysia, 43400, Serdang, Selangor

^2^Department of Biological Function and Engineering, Graduate School of Life Science and System Engineering, Kyushu Institute of Technology, 2-4 Hibikino-cho, Wakamatsu-ku, Fukuoka 808-0196, Japan

^3^Department of Bioprocess Technology, Faculty of Biotechnology and Biomolecular Sciences, Universiti Putra Malaysia, 43400 UPM Serdang, Selangor.

^4^Advanced Microbiological Functions Research Group, Frontier Research Labs., Institute for Innovation, Ajinomoto, 1-1 Suzuki-cho, Kawasaki-ku, Kawasaki, Japan;

***Corresponding author**: Mohd Huzairi Mohd Zainudin

Laboratory of Sustainable Animal Production and Biodiversity, Institute of Tropical Agriculture and Food Security, Universiti Putra Malaysia, 43400 UPM Serdang, Selangor, Malaysia;

Tel: +60397694287; Fax: +60389381612; e-mail: [mohdhuzairi@upm.edu.my](mailto:mohdhuzairi@upm.edu.my)

1 2 3


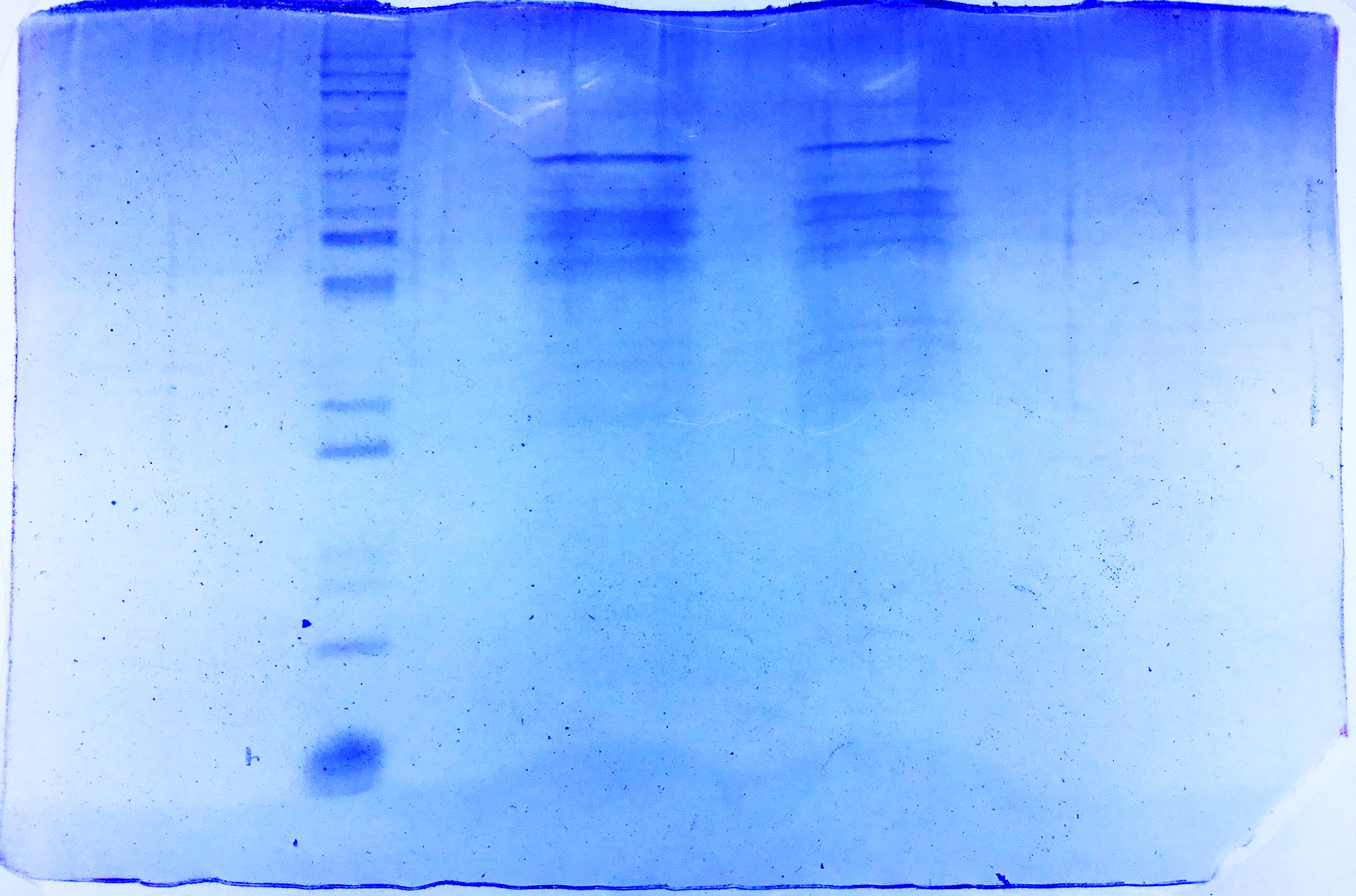


Fig S1. Protein profile of the *T. fusca* UPMC 901 crude enzyme preparation. Lane 1: 150kDa Marker; Lane 2 and 3- Duplicates of extracellular proteins of CMC cell-free culture supernatant.

Fig. S2. The endoglucanase activity of the crude enzyme visualized by using SDS gel containing 0.5% (w/v) of CMC. Lane 1 and 2 – endoglucanase activity (CMCase) of duplicates of extracellular proteins cell-free culture supernatant.
